# Seroprevalence of Immunoglobulin M and G antibodies to severe acute respiratory syndrome coronavirus 2 infection in Iwo, Nigeria

**DOI:** 10.1186/s12879-025-11850-1

**Published:** 2025-10-29

**Authors:** Yewande Tolulope Nejo, Oladipo Olarinre Oladosu, Matthew Idowu Olatubi, Abimbola Adetokunboh Owoseni, Samuel Olatunde Dahunsi

**Affiliations:** 1 Microbiology Programme, College of Agriculture, Engineering and Science, Bowen University, Iwo, Nigeria; 2 Pure and Applied Biology Programme, College of Agriculture, Engineering and Science, Bowen University, Iwo, Nigeria; 3 Nursing Science Programme, College of Health Sciences, Bowen University, Iwo, Nigeria; 4 Department of Virology, Faculty of Basic Medical Sciences, College of Medicine, University of Ibadan, Ibadan, Nigeria

**Keywords:** Seroprevalence, SARS-CoV-2, COVID-19, IgM and IgG, Nucleocapsid protein, Iwo nigeria

## Abstract

**Background:**

In many countries, the preliminary stage of the COVID-19 pandemic has reached its climax and serological studies are extremely important in managing public health responses to the COVID-19 pandemic caused by SARS-CoV-2. The Nucleocapsid protein, a highly immunogenic protein, found only in infected individuals is important in SARS-CoV-2 infection. This study was therefore designed to determine the seroprevalence of SARS-CoV-2 IgM and IgG antibodies to nucleocapsid proteins in Iwo, Nigeria, thereby providing information on the spread of the infection.

**Methods:**

Five millilitres of blood were collected from 360 individuals visiting four selected hospitals in Iwo, Nigeria. Socio-demographic and clinical data of the participants were obtained through a structured questionnaire. SARS-CoV-2 IgM and IgG antibodies against the nucleocapsid protein were detected from plasma samples using commercially available Enzyme-linked Immunoassay kits (Monobind Inc, USA) according to the manufacturer’s instructions. Statistical analyses were performed using SPSS software version 21 at *P* < 0.05 for statistical significance.

**Results:**

Out of the tested samples, 37 and 165 were positive for IgM and IgG antibodies giving a prevalence of 10.3% [95% CI: 7.5% − 13.6%] and 45.8% [95% CI: 40.8% − 50.8%], respectively. In total, 184 samples tested positive for SARS-CoV-2 antibodies giving an overall prevalence of 51.1% [95% CI: 45.8% − 56.1%]. Infection was associated with age; older population (35–90 years), fever, cough and muscle pain (*p* < 0.05). SARS-CoV-2 IgM and IgG antibodies were detected among 11.8% and 42.6% of COVID-19 vaccine recipients, respectively.

**Conclusions:**

High seroprevalence of SARS-CoV-2 antibodies to nucleocapsid proteins were detected among the study population. In particular, is detecting IgM antibodies among those who have received COVID-19 vaccines. This reveals a high level of recent and past exposure to SARS-CoV-2 virus infection and continuous spread within the community.

## Background

Coronavirus disease 2019 (COVID-19), a highly infectious respiratory disease is caused by severe acute respiratory syndrome coronavirus 2 (SARS-CoV-2) [1]. The COVID-19 pandemic first emerged in Wuhan, Hubei province, China in December 2019 causing an outbreak of unusual viral pneumonia. The COVID-19 outbreak was declared a Public Health Emergency of International Concern (PHEIC) on 30th January 2020 and a pandemic on 11th March 2020 by the World Health Organization (WHO) [2]. SARS-CoV-2 poses a critical threat to public health and medical institutions and infection is still spreading. As of 14th July 2024, the disease has spread globally to 228 countries and territories with 775,686,716 confirmed cases and 7,054,093 reported deaths [2].

Coronavirus is an RNA virus consisting of positive-sense single-stranded RNA of approximately 27–32 kb. The genome of SARS-CoV-2 has higher similarity (over 96% identity at the whole-genome level) to two bat-derived coronavirus strains than to other known human coronaviruses, and pangolins have been reported to be the intermediate host of SARS-CoV-2 [3]. Four different genera (*Alphacoronavirus*, *Betacoronavirus*, *Gammacoronavirus* and *Deltacoronavirus*) in the subfamily *Coronavirinae* of the *Coronaviridae* family are responsible for coronavirus infections in humans and other vertebrate species. SARS-CoV-2 belongs to the genus *Betacoronavirus* which includes two viruses [severe acute respiratory syndrome coronavirus (SARS-CoV) and Middle East Respiratory Syndrome coronavirus (MERS-CoV)] that have previously caused epidemics. Infected hosts exhibit different clinical courses, ranging from asymptomatic to severe symptoms in their respiratory, digestive, and genital organs [4]. Six known coronaviruses typically cause infection in humans before the SARS-CoV-2 pandemic. Among these, coronavirus 229E, OC43, NL63, and HKU1 generally cause mild cold-like symptoms, whereas SARS-CoV in 2003, and MERS-CoV in 2012, caused severe respiratory diseases such as pneumonia and death [5, 6].

Three possible transmission phases of SARS-CoV-2 infection between individuals have been identified, and these include symptomatic, pre-symptomatic and asymptomatic transmission phases. Symptomatic transmission occurs in infected persons who have already developed signs and symptoms of the infection. The reported symptoms of COVID-19 include cough, fever, difficulty in breathing and diarrhoea. Pre-symptomatic transmission occurs in infected persons during the incubation period (5–14 days) of the virus i.e., between the time of infection and the manifestations of symptoms. However, asymptomatic transmission occurs in infected persons without any symptoms of infection but transmits the virus to another person [7].

Developing nations including Nigeria were not left out in the fight against the COVID-19 pandemic. In Nigeria, the first identified case of COVID-19 was diagnosed in an international immigrant on 27th February 2020 [8]. Nigeria, the most populous country in Africa and with an estimated 226 million inhabitants [9] has recorded 267,188 confirmed cases of COVID-19 with 3,155 reported deaths as of 13th April 2024 [10]. Only 5,915,616 individuals were reported to have been tested as of 26th February 2023 [11] after which information about COVID-19 testing was no more available.

There is well-established evidence of community transmission of SARS-CoV-2 in Nigeria and 71% of confirmed cases have no known exposure history [12]. Hence, contact tracing of high-risk individuals is not feasible anymore as many people would have been infected without knowing. Due to the lack of specific antiviral treatments and the burden of clinical treatment, thousands of severe cases have died globally. It, therefore becomes imperative to focus on population-based testing rather than testing only symptomatic patients and travellers, especially in a town like Iwo, Nigeria where there are no testing centres and many people do not believe in the existence of COVID-19.

Every person that is exposed to SARS-CoV-2 will develop antibodies to the virus, hence serological testing is more important to monitor the pandemic progression rather than molecular testing alone, thereby giving broader estimates of infected individuals [13]. Moreover, Immunoglobulin M (IgM) and Immunoglobulin G (IgG) antibody testing can be used to provide information on both acute infection and past exposure respectively thereby informing public health response to COVID-19 [7]. Furthermore, as the world moves through the vaccine and variant era, seroepidemiology findings are increasingly important to track the spread of infection, identify excessively affected groups and progression towards herd immunity.

Out of the sixteen non-structural and four structural proteins encoded by the SARS-CoV-2 genome, the coronavirus spike (S) proteins have received the most attention in research because of their significance in the viral life cycle [14, 15],. Spike proteins have been targeted in the development of vaccines as well as in the majority of serological diagnoses of SARS-CoV-2, but are unable to distinguish between antibodies produced in response to a previous infection and those produced in response to vaccination. Nevertheless, numerous SARS-CoV-2 proteins, particularly the Nucleocapsid protein (N protein), which is only detected in infected individuals, are just as crucial to the virus’s life cycle. In addition to serving as the basis for the packaging of the viral RNA genome into the ribonucleotide complex (RNP) and assembly into virus particles, the N protein is also the most prevalent protein in virions and a highly immunogenic antigen [16, 17]. It is therefore important to conduct a serological survey of SARS-CoV-2 infection through nucleoprotein antibody detection in Iwo, Nigeria, thereby revealing the level of exposure to the infection.

## Materials and methods

### Study design and participants

This is a cross-sectional study conducted in Iwo, Osun State, Nigeria, and approval was received from the Osun State Ministry of Health Research Ethics Committee with the reference number: OSHREC/PRS/569T/238. The participants for this study were enrolled from three different hospitals in Iwo town and these include a State Hospital (*n* = 124), a Tertiary University Hospital (*n* = 101) and two Private Hospitals (*n* = 135). The selection of these hospitals encompasses all categories of individuals living in Iwo, Osun State, ranging from the poor to the rich, healthy to individuals with clinical conditions, thereby forming a representative sample of the population.

Participants were recruited using a convenience sampling technique and they include persons 18 years and older. These individuals visit the selected hospitals either for routine medical check-up or for treatment purposes. Participants were allowed to enrol whether or not they had developed COVID-19 symptoms since the onset of the pandemic. Individuals who have either received or not received COVID-19 vaccines were also allowed to participate in the study. Informed consent was obtained from all the participants and those who did not give their consent or are younger than 18 years were not included in the study. Participants were recruited between April and June 2022.

### Sample collection

During this study, 5mLs of venous blood were collected from a total of 360 participants using venepuncture by trained laboratory technicians. The samples were dispensed in labelled Ethylene diamine tetra-acetic acid (EDTA) bottles. Socio-demographic and clinical data of the participants were obtained through a structured questionnaire. The samples collected were transported on ice to the laboratory. Upon centrifugation of the blood samples at 1000 x *g* for 20 min, plasma samples were separated from the whole blood and transferred into pre-labelled cryovials. The plasma and blood cells were stored in the freezer at −40 °C until further analysis.

### Detection of SARS-CoV-2 antibodies

Samples were analysed using Accubind ELISA test kits (Monobind Inc., Lake Forest, CA 92630 USA) for the qualitative detection of IgM and IgG antibodies to SARS-CoV-2 nucleocapsid protein according to the manufacturer’s instruction. The sensitivity of the IgM and IgG test kits is at least 98.3% and 97.6% positive percent agreement, respectively, while both assays have a 100% Specificity. The absorbance of all the samples were read using DNM-9602 Microplate Reader (Drawell International Technology Limited, China). The Cut-Off Value of the Cut-Off Control was calculated and used to ascertain the positivity or the negativity of the samples for both IgM and IgG assay according to the manufacturer’s instruction. The tests utilise the nucleocapsid protein of SARS-CoV-2, and antibodies against any component of the spike protein were not detected. Hence, a positive result verifies a recent or past infection with SARS-CoV-2. Samples from patients who have received the SAR-CoV-2 spike protein vaccine but have not been exposed to the live virus will not react with the test.

### Data management and statistical analyses

Data obtained through questionnaire administration and the results of the laboratory analysis were entered into Statistical Package for Social Sciences (SPSS) software version 21 for analyses. Descriptive and inferential statistics were done. The chi-square test was used to estimate the degree of correlation between the variables in this study with *p-values* of < 0.05 considered statistically significant.

## Results

### Socio-demographic characteristics and Seroprevalence of SARS-CoV-2 antibodies among the study participants

Out of the participants tested in this study (*n* = 360), a total of 184 individuals were positive for SARS-CoV-2 antibodies indicating an overall seroprevalence of 51.1% [95% CI: 45.8% − 56.1%] (Table 1). All the participants were Nigerians and the majority (90.1%) were of the Yoruba ethnic stock. The age of the participants ranged from 18 to 90 years with a mean value of 31.2 ± 14.8, but the highest prevalence of SARS-CoV-2 antibodies was significantly (*P* = 0.038) found among the older ones (35–90 years). Although more female participants (76.7%) were recruited, a higher prevalence of SARS-CoV-2 infection was observed among the males (57.1%) but not statistically significant (*P* = 0.207). More than half of the recruits (54.2%) were married, but only 32% had children living with them. The highest prevalence of SARS-CoV-2 antibodies was significantly detected among individuals with the highest number of children (*n* = 5 to 7; 78.3%). About 59% of the participants have attained tertiary educational level and quite a number of the participants were without routine jobs (46.4%). Some of the workers (9.2%) had low incomes (less than ₦18,000.00) and many (53.9%) did not know their average monthly income, especially the self-employed. Though a few of the participants (1.9%) have travelled outside the country since the COVID-19 pandemic, a higher prevalence of infection (71.4%) was detected among them. Infection with SARS-CoV-2 was not associated with marital status, marriage type, education, employment status and monthly income (Table 1).

**Table 1:** Socio-demographic characteristics and overall prevalence of SARS-CoV-2 antibodies among the study participants

Variables (*P* values in bold)	No. Enrolled (%)	No. positive (%)	Variables (*P* values in bold)	No. Enrolled (%)	No. positive (%)
**State of Residence (0.198)**			*Number of Children (0.012)		
Ekiti	1 (0.3)	1(100)	1–2	57 (49.1)	24 (42.1)
Lagos	30 (8.3)	20 (66.7)	3–4	36 (31.0)	17 (47.2)
Ogun	5 (1.4)	2 (40.0)	5–7	23 (19.8)	18 (78.3)
Ondo	2 (0.6)	0 (0.0)	**Education (0.394)**		
Osun	282 (78.3)	137 (48.6)	None	19 (5.3)	11 (57.9)
Oyo	20 (5.6)	13 (65.0)	Primary	19 (5.3)	13 (68.4)
Others	20 (5.6)	11 (55.0)	Secondary	102 (28.3)	49 (48.0)
**Ethnic Stock (0.135)**			Tertiary	212 (58.9)	105 (49.5)
Yoruba	326 (90.6)	163 (50.0)	Quaranic	3 (0.8)	2 (66.7)
Others	33 (9.2)	21 (63.6)	No Response	5 (1.4)	4 (80.0)
*Age (Years) (0.038)			**Employment (0.475)**		
18–24	149 (41.4)	71 (47.7)	Unemployed	167 (46.4)	78 (46.7)
25–34	104 (28.9)	46 (44.2)	Employed	59 (16.4)	32 (54.2)
35–44	42 (11.7)	23 (54.8)	Self Employed	128 (35.6)	71 (55.5)
45–54	33 (9.2)	23 (69.7)	No Response	6 (1.7)	3 (50.0)
55–90	32 (8.9)	21 (65.6)	**Monthly Income (₦) (0.364)**		
**Gender (0.207)**			Less than 18,000	33 (9.2)	21 (63.6)
Male	84 (23.3)	48 (57.1)	18,000–50,000	20 (5.6)	12 (60.0)
Female	276 (76.7)	136 (49.3)	51,000–100,000	18 (5.0)	9 (50.0)
**Marital Status (0.886)**			101,000–200,000	3 (0.8)	1 (33.3)
Single	158 (43.9)	80 (50.6)	201,000–500,000	2 (0.6)	2 (100.0)
Married	195 (54.2)	101 (51.8)	Don’t Know	194 (53.9)	91 (46.9)
Widow	7 (1.9)	3 (42.9)	Not Applicable	90 (25.0)	48 (53.3)
**Marriage Type (0.987)**			*Travel History (0.004)		
Monogamy	140 (38.9)	72 (51.4)	Within State	116 (32.2)	45 (38.8)
Polygamy	62 (17.2)	32 (51.6)	Outside State	237 (65.8)	134 (56.5)
Unmarried	158 (43.9)	80 (50.6)	Outside Nigeria	7 (1.9)	5 (71.4)
**Total**	360 (100.0)	184 (51.1)	**Total**	360 (100.0)	184 (51.1)

*Significant *P* values

### Clinical information and prevalence of SARS-CoV-2 IgM and IgG antibodies among the study participants

Table 2 shows the seroprevalence of SARS-CoV-2 IgM and IgG antibodies in relation to the clinical information of the study participants. In all, a total of 37 samples tested positive for IgM antibodies giving a prevalence of 10.3% [95% CI: 7.5% − 13.6%] while 165 tested positive for IgG antibodies giving a prevalence of (45.8%) [95% CI: 40.8% − 50.8%]. Out of these values, 19 (5.3%) tested positive for SARS-CoV-2 IgM antibodies only; 147 (40.8%) tested positive for IgG antibodies only; and 18 (5.0%) tested positive for both IgM and IgG antibodies.

**Table 2:** IgM and IgG Seroprevalence of SARS-CoV-2 based on the clinical history of the study participants

Variables (*P* values in bold)	No. Enrolled (%)	IgM Positive (%)	IgG Positive (%)	Overall positive (%)
**Underlying Disease**		**0.169**	**0.167**	**0.235**
Yes	3 (0.8)	1 (33.3)	3 (100.0)	3 (100.0)
No	357 (99.2)	36 (10.1)	162 (45.4)	181 (50.7)
**Fever**		**0.597**	**0.057**	*0.032
Yes	94 (26.1)	11 (11.7)	51 (54.3)	57 (60.6)
No	266 (73.9)	26 (9.8)	114 (42.9)	127 (47.7)
**Cough**		**0.309**	*0.010	*0.023
Yes	57 (15.8)	8 (14.0)	35 (61.4)	37 (64.9)
No	303 (84.2)	29 (9.6)	130 (42.9)	147 (48.5)
**Chills**		**0.333**	**0.155**	**0.061**
Yes	33 (9.2)	5 (15.2)	19 (57.6)	22 (66.7)
No	327 (90.8)	32 (9.8)	146 (44.6)	162 (49.5)
**Nasal Congestion**		**0.167**	**0.283**	**0.191**
Yes	43 (11.9)	7 (16.3)	23 (53.5)	26 (60.5)
No	317 (88.1)	30 (9.5)	142 (44.8)	158 (49.8)
**Sore throat**		**0.959**	**0.150**	**0.198**
Yes	67 (18.6)	7 (10.4)	36 (53.7)	39 (58.2)
No	293 (81.4)	30 (10.2)	129 (44.0)	145 (49.5)
**Nausea**		**0.109**	**0.289**	**0.468**
Yes	37 (10.3)	1 (2.7)	20 (54.1)	21 (56.8)
No	323 (89.7)	36 (11.1)	145 (44.9)	163 (50.5)
**Loss of taste & smell**		**0.420**	**0.802**	**0.631**
Yes	27 (7.5)	4 (14.8)	13 (48.1)	15 (55.6)
No	333 (92.5)	33 (9.9)	152 (45.6)	169 (50.8)
**Muscle Pain**		**0.589**	**0.240**	*0.021
Yes	91 (25.3)	8 (8.8)	51 (56.0)	56 (61.5)
No	269 (74.7)	29 (10.8)	114 (42.4)	128 (47.6)
**Shortness of breath**		*0.018	**0.890**	**0.543**
Yes	19 (5.3)	5 (26.3)	9 (47.4)	11 (57.9)
No	341 (94.7)	32 (9.4)	156 (45.7)	173 (50.7)
**Fatigue**		**0.173**	**0.087**	**0.125**
Yes	69 (19.2)	4 (5.8)	38 (55.1)	41 (59.4)
No	291 (80.8)	33 (11.3)	127 (43.6)	143 (49.1)
**GIT Upset**		**0.130**	**0.541**	**0.892**
Yes	19 (5.3)	0 (0.0)	10 (52.6)	10 (52.6)
No	341 (94.7)	37 (10.9)	155 (45.5)	174 (51.0)
**Symptoms**		**0.735**	*0.002	*0.002
Symptomatic	156 (43.3)	17 (10.9)	86 (55.1)	94 (60.3)
Asymptomatic	204 (56.7)	20 (9.8)	79 (38.7)	90 (44.1)
**Symptom’s Intensity**		**0.409**	*0.006	*0.009
Mild	144 (40.0)	17 (11.8)	78 (54.2)	86 (59.7)
Severe	12 (3.3)	0 (0.0)	8 (66.7)	8 (66.7)
No Symptoms	204 (56.7)	20 (9.8)	79 (38.7)	90 (44.1)
**Total**	**360 (100.0)**	**37 (10.3)**	**165 (45.8)**	**184 (51.1)**

*Significant *P* values

The highest overall prevalence of SARS-CoV-2 antibodies was significantly found among those with symptoms of infection (*P* = 0.002) and those who experienced the severity of symptoms (*P* = 0.009). The most frequently reported symptoms were fever (26.1%), muscle pain (25.3%), fatigue (19.2%), sore throat (18.6%) and cough (15.8%). Overall positivity to SARSCoV-2 antibodies was associated with people who reported having fever (*P* = 0.032), cough (*P* = 0.023) and muscle pain (*P* = 0.021). However, shortness of breath was significantly associated with the infection among those who tested positive for IgM antibodies (*P* = 0.018).

### COVID-19 history and prevalence of SARS-CoV-2 IgM and IgG antibodies among the study participants

Table 3 shows that only 13.1% of the study participants reported being previously screened for COVID-19, and although the majority (92.2%) knew about COVID-19 vaccination, only 18.9% reported receiving either one (8.6%) or two (10.3%) doses of the vaccine. Some of the participants (27.9%) gave reasons for not receiving the vaccine while more than half of the population (53.3%) gave no reasons. SARS-CoV-2 IgM and IgG antibodies were detected among 11.8% and 42.6% of those who reported to have received the COVID-19 vaccine, respectively.

**Table 3:** Seroprevalence of SARS-CoV-2 IgM and IgG antibodies based on COVID-19 history

Variables (*P* values in bold)	No. Enrolled (%)	IgM positive (%)	IgG Positive (%)	Overall Positive (%)
**COVID-19 Exposure**		**0.187**	**0.663**	**0.588**
Yes	3 (0.8)	1 (33.3)	1 (33.3)	2 (66.7)
No	357 (99.2)	36 (10.1)	164 (45.9)	182 (51.0)
**COVID-19 Testing**		**0.930**	**0.266**	**0.749**
Yes	47 (13.1)	5 (10.6)	18 (38.3)	23 (48.9)
No	313 (86.9)	32 (10.2)	147 (47.0)	161 (51.4)
**COVID Vaccine Knowledge**		**0.062**	**0.948**	**0.606**
Yes	332 (92.2)	37 (11.1)	152 (45.8)	171 (51.5)
No	28 (7.8)	0 (0.0)	13 (46.4)	13 (46.4)
**COVID Vaccination**		**0.654**	**0.558**	**0.737**
Yes	68 (18.9)	8 (11.8)	29 (42.6)	36 (52.9)
No	292 (81.1)	29 (9.9)	136 (46.6)	148 (50.7)
**Vaccine Dosage**		**0.790**	**0.468**	**0.238**
1	31 (8.6)	3 (9.7)	11 (35.5)	13 (41.9)
2	37 (10.3)	5 (13.5)	18 (48.6)	23 (62.2)
No vaccine	292 (81.1)	29 (9.9)	136 (46.6)	148 (50.7)
**Reasons for Vaccine Decline**		*0.022	**0.114**	**0.200**
Pregnant/Breastfeeding	2 (0.6)	0 (0.0)	2 (100.0)	2 (100.0)
Fear & Misconception	56 (15.6)	9 (16.1)	30 (53.6)	33 (58.9)
Ignorance/No access to vaccine centres	39 (10.9)	1 (2.5)	22 (56.4)	22 (56.4)
On TB Medication	3 (0.8)	2 (66.7)	2 (66.7)	2 (66.7)
No reasons	192 (53.3)	17 (8.9)	80 (41.7)	89 (46.4)
Vaccinated	68 (18.9)	8 (11.8)	29 (42.6)	36 (52.9)
**Total**	**360 (100.0)**	**37 (10.3)**	**165 (45.8)**	**184 (51.1)**

*TB* Tuberculosis

*Significant *P* values

## Discussion

Variations exist in the epidemiology of SARS-CoV-2 infection globally. The morbidity and mortality observed in Nigeria have been extremely low compared with developed countries and even other African countries [18, 19]. The result of this study revealed an overall SARS-CoV-2 seroprevalence of 51.1% among the study participants. This study is the first serological investigation of SARS-CoV-2 in Iwo, Nigeria and the data was collected in the year 2022 after the third wave of coronavirus. At this time, Nigerians were fully back to their routine businesses and way of life following the easing of the lockdown. The high prevalence recorded in this study correlates with the prevalence of 53.5% reported by Chechet et al. [20] from the rural site of Kaduna, Nigeria. However, this study’s seroprevalence is higher than the prevalence recorded in some previous studies in Nigeria [21, 22] but lower than the 78.9% and 66.7% reported by Kolawole et al. [23], and Onifade et al. [24] respectively.

The prevalence of SARS-CoV-2 obtained from this study is one of the highest recorded in Nigeria. It shows both individuals who have been previously exposed (IgG positive; 45.8%) as well as those with recent infections (IgM positive; 10.3%) at the time of sampling. Using the result of both assays for serosurvey shows the true prevalence of exposure to the infection, and the IgM positive results revealed that the transmission of SARS-CoV-2 infection is still ongoing among the population. High exposure to SARS-CoV-2 infection as reflected by the high prevalence among these individuals could be attributed to a low level of adherence to prevention and control measures such as the use of nose masks, washing of hands, physical distance etc. in the community. Many individuals in this part of the country also do not believe in the existence of COVID-19 and so do not adhere to these measures. Additionally, in a low-income country such as Nigeria, several factors such as ignorance, financial concerns, lack of personal hygiene and mistrust of leaders are responsible for the inability to enforce these precautionary measures on the population.

Several serological studies suggest that younger adults particularly those aged under 35 years often have high cumulative rates of SARS-CoV-2 infection in the community [25–27], yet other evidence suggests that susceptibility to the infection generally increases with age [7, 27]. This latter pattern was observed in this study as participants with older age (35 years and older) were associated with a higher prevalence of SARS-CoV-2 antibodies with the peak prevalence among the age group 45–54 years. This study also corroborates the results of other studies [28, 29].

The prevalence of SARS-CoV-2 antibodies observed among the male participants in this study was higher than that of the females but not statistically significant. However, some studies have shown that the male gender is a risk factor for a severe SARS-CoV-2 infection [29–31]. Different theories from scientists have explained the reasons for this discovery, and these include variations in sex-mediated immunological responses, higher susceptibility to bacterial and viral infections in men, and differential expression of angiotensin-converting enzyme 2 (ACE2) in both genders. According to Vikse et al. [32], SARS-CoV-2 finds refuge in male testicles, prolonging the virus elimination.

Marital status and type of marriage were not associated with SARS-CoV-2 infection in this study but the infection was associated with the increasing number of children living in a family, with the highest prevalence found among families having between five and seven children. It is expected that the prevalence of SARS-CoV-2 should be higher among children than adults because of the way children associate with themselves without restriction. Similarly, prevalence increases as the number of people in close contact with people who also mix daily with others in the community increases. Nevertheless, this study did not capture children as part of the study population, but having more children in a household could increase the risk of transmitting or acquiring such infection as a result of closer and more prolonged interactions among family members.

It is generally believed that travelling especially with public transportation in a resource-poor country like Nigeria increases the risk of contracting respiratory infections like SARS-CoV-2 because of the inability to ensure physical distancing. The travel history of participants during the pandemic years was associated with the seroprevalence of SARS-CoV-2, although the type of transportation used was not included. Many of the participants (65.8%) moved from state to state despite the lockdown, however, the highest prevalence of the infection (71.4%) was detected among the few people who had travelled outside the country since the pandemic.

More than half of the study participants (56.7%) had no symptoms related to COVID-19 at the time of sample collection, but 44.1% of these individuals tested positive for SARS-CoV-2 antibodies. Although some studies [33, 34] have demonstrated the possibilities of asymptomatic transmission of SARS-CoV-2 infection, Utulu et al. [34] revealed a higher significance of symptoms such as coughing and sneezing in infection transmission. Similarly, symptomatic individuals (i.e. those with fever, cough, chills and muscle pain) and those with severity of symptoms in this study had a significantly higher prevalence of SARS-CoV-2 antibodies. These individuals especially those with recent infection (IgM positive) may serve as sources of continuous transmission of the infection in the community.

A few participants have also had a previous COVID-19 test, and the high prevalence of participants who never had a COVID-19 test in this study is consistent with the report of Al-Mustapha et al. [35] and Onifade et al. [24]. This showed that the true prevalence of the infection is possibly not known in Nigeria, and this could be due to challenges facing the health system in the country which has resulted in inadequate services. Lack of access to health care facilities where these tests are being conducted amongst others is one of the factors affecting the mass testing of individuals.

Although the majority of individuals tested in this study were knowledgeable about COVID-19 and its vaccine, only 18.9% had received one of the two available vaccines (AstraZeneca or Pfizer) in Iwo Nigeria at the time of sampling. Out of those who received the vaccines, 8.6% and 10.3% reported to have received 1 and 2 doses, respectively. Our study revealed a great awareness and information dissemination about vaccination as a means of prevention and control of the infection but observed a low turnout of vaccination among the people as many chose not to receive it even at no extra cost. When the recruits were asked about their refusal to be vaccinated, more than half gave no reasons while 15.6% reported being afraid of the side effects and misconceptions about the vaccine due to bad publicity. Other reasons given by the respondent included pregnancy, breastfeeding, lack of access to vaccination centres, being on long-term medications like tuberculosis drugs and ignorance of the vaccine. From the data collected from these study participants, it shows that a lot of them do not have the correct information about COVID-19 vaccination. Therefore, accurate and on-time dissemination of information to the public about who and when to receive the vaccine is very important, especially through various social media outlets. Immediate response by the different healthcare professionals and relevant bodies in different languages and simplicity to negate the wrong information being circulated to the public will go a long way to addressing the issue of bad publicity.

Nonetheless, it was observed that out of the individuals who claimed to have received either one or two doses of the COVID-19 vaccine, more than half still tested positive for SARS-CoV-2 antibodies. This is surprising as this study tested for antibodies to SARS-CoV-2 nucleocapsid protein which is to be found only in people who are exposed to the virus. Antibodies to spike proteins are rather found in vaccinated individuals. However, the detection of nucleocapsid protein antibodies among this group of individuals could be as a result of exposure to the virus before the individuals were vaccinated, although this could not be ascertained in this study. Nevertheless, it was still observed that 8 (11.8%) of these vaccinated individuals tested positive for IgM antibodies which is an indicator of a recent or acute infection. These individuals reported to have received the vaccine at least 4 months before their blood was collected for the test except two individuals who received it one or two months before enrolment for the study. It is expected that the vaccine received should stimulate neutralising antibodies against subsequent exposure to SARS-CoV-2 infection but the result showed that despite being vaccinated, individuals still got infected with the virus. However, the small sample size and inability to quantify the detected antibodies and determine if they were neutralising antibodies are the major limitations of this study.

## Conclusion

A high seroprevalence of SARS-CoV-2 IgM and IgG antibodies among the study population was observed which shows a high level of both recent and past exposure to the infection and evidence of continued transmission among the population. The prevalence of the infection was associated with age, increasing number of children in a household, travel history of the participants, symptomatic persons, and severity of symptoms. Although COVID-19 was recently declared to no longer be a public health emergency, it is, therefore, important to continue to emphasise public health measures and monitor the progression of the infection to sustain the strategy for infection control and prevent a resurgence of severe COVID-19 cases in the future. Additionally, surveillance of the circulating strains is advocated continually to curtail future pandemics that might result from the emergence of virulent novel strains. The question of the effectiveness of COVID-19 vaccines is raised in the study as those reported to have been vaccinated some months before screening still tested positive for SARS-CoV-2 IgM antibodies which is an indication of recent infection. Thus, it is necessary to conduct post-vaccination anti-SARS-CoV-2 neutralising antibody screening to determine the effectiveness of COVID-19 vaccines in controlling the infection and to understand how long protection from these vaccines lasts.

## Data Availability

All data generated or analysed during this study are included in this published article.
